# Vaccines Attitudes, Concerns, and Information Sources Reported by Parents of Young Children among North Palestinian Parents

**DOI:** 10.1155/2020/8028172

**Published:** 2020-10-31

**Authors:** Issa Alawneh, Abdulkareem Saymeh, Ahmad Yasin, Maysa Alawneh, Hossam Al-Tatari

**Affiliations:** ^1^Department of Pediatrics, Najah University Hospital, Nablus, State of Palestine; ^2^Department of Pediatrics, Hamad Medical Corporation, Doha, Qatar; ^3^The Heart Medical Center, Al Ain, UAE

## Abstract

Parental acceptance of routine childhood immunization is critical to protecting children's health, as high vaccination-coverage rates lead to decreased rates of vaccine-preventable diseases. However, to communicate effectively with parents about vaccines and vaccine-preventable diseases, it is necessary to assess their vaccine-related attitudes and concerns continually. Recently the Palestine Ministry of Health has recorded epidemics of measles and mumps. Poor compliance with vaccination has been attributed to multiple factors including physician inadequacy advocating for vaccination and public mistrust of vaccinations. As a result, this study was conducted to describe the vaccine-related attitudes, concerns, and information sources of North Palestinian parents of young children. A cross-sectional survey was conducted involving parents visiting emergency departments and primary health care centers from different North Palestinian hospitals and centers. 480 surveys were eligible and analyzed. The surveys revealed that although parental confidence in vaccine safety is high, several vaccine-related concerns, such as pain from vaccine administration and the number of vaccines given at once, were common among parents of young children. To maintain and improve the success of childhood vaccines in preventing disease, a holistic approach is needed to address parents' concerns in an ongoing manner. Listening and responding in ways and with resources that address specific questions and concerns could help parents make more informed vaccination decisions.

## 1. Introduction

Parental acceptance of routine childhood immunization is essential to protecting and saving children's health. A study showed that high coverage of recommended vaccines in the United States has resulted in a reduction in the incidence of greater than 99% for many vaccine-preventable diseases [1]. Over the years, some clusters of people, parents, and schools used to reject using the vaccines as well as preventing others from taking them using various campaigns, beliefs, and thoughts not linked to evidence-based medicine. So we can say that concern over the safety or necessity of vaccination is not a new phenomenon [2].

World Health Organization (WHO) has recorded epidemics of measles and mumps. Similarly, the Palestinian Ministry of Health has recorded such epidemics recently in Palestine [3]. In the Unites States, the potentials for new epidemics of vaccine-preventable diseases mandates the need for effective communication regarding accurate information on vaccination to parents who are the cornerstone in the children's vaccination process [1].

Parental concerns that vaccines are not safe for their children has been studied in recent years and linked to several factors. These factors include the number of vaccines in the recommended childhood immunization schedule [4]. The presence of false and conflicting information regarding vaccines safety most likely obtained from antivaccines contents through the Internet stressing on the point that it is impossible to regulate the information reaches parents searching the Internet [5, 6].

Published theories linked vaccines to chronic medical disease and disabilities, such as the study that linked vaccines' use with the subsequent development of autism [7].

The vaccination program in Palestine is undertaken by the Palestinian Authority: Ministry of Health (MOH) since 1994. The vaccination process is mainly administered at primary health care centers. It is mandatory for all children as per the recommendations of the Ministry of Health. However, parents can decline vaccination to their children. In such case, huge efforts will put forward to explain the importance of taking the vaccination. That will explain the benefits as well as vaccine safety to the parents in multiple sessions if needed [8].

Optimistically, the overall success rate of the Palestinian childhood vaccination program can help perpetuate these concerns. Because the overall vaccine-preventable disease rates are low in our country, parents who have no personal experience with vaccine-preventable diseases might focus their attention on the perceived risks of vaccines instead of their well-documented benefits [9].

Parents who question the necessity or safety of vaccines for infants may ultimately choose to either decline or delay vaccination, which will leave their children vulnerable to disease. In addition, an unvaccinated child in a community threatens the health of those who are too young to be vaccinated and those for whom immunization is contraindicated.

To communicate effectively with parents about vaccines and vaccine-preventable diseases, it is necessary to assess their vaccine-related attitudes and concerns continually. We conducted this study to describe the vaccine-related attitudes, concerns, and information sources of Palestinian parents. This is the first study to be conducted in Palestine. A similar study was conducted in the United Arab Emirates (UAE) [10], and internationally by the 2009 Health Styles Survey study [11].

## 2. Objectives

This study was conducted to describe the vaccine-related attitudes, concerns, and information sources of North Palestinian parents.

## 3. Methods

After An-Najah National University Institution Review Board (IRB) approval and obtaining the permissions from Palestinian MOH, in the period between August 2014 and October 2017, daily visits were conducted to parents visiting pediatric emergency departments and primary health care centers involving five major cities in North Palestine including Nablus, Jenin, Tulkarem, Tubas, and Qalqilia.

The two principal investigators served as interviewers. They were starting by thoroughly explaining the research and its purpose for participants and then taking verbal consent.

Then a checklist questionnaire, based on the inclusion and exclusion criteria, was completed.

After that, a cross-sectional survey was conducted (Annex). It consists of thirteen multiple choice items. The survey was used in the United Arab Emirates (UAE) study in 2015 at Tawam Hospital [10]. It was reevaluated and validated by the ethics committee at Najah National University Hospital, Palestine.

 
*Inclusions criteria*. Male or female, at least 18 years of age, married, and at least have one dependent child.
 
*Exclusion criteria*. Less than 18 years of age, single, married but does not have children.

## 4. Results

**Figure 1:**
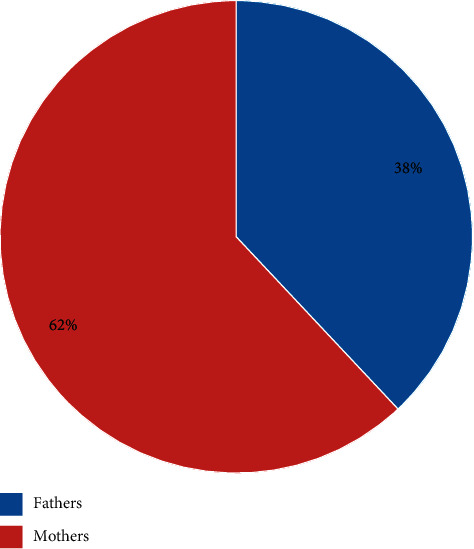
Parents surveyed.

The response rate was 94%. Six hundred and fifty-nine surveys were collected. Four hundred and eighty surveys were eligible and analyzed. The majority of parents surveyed were female (Figure 1).

As Table 1 shows, 16% of the surveyed parents were between 18 and 24 years old. Most of them, about 56%, were between 25 and 34 years old. The remaining 28% of them were above 34 years old. When it comes to the educational level, about half of the parents completed a university degree.

Almost all the surveyed parents, 97% of them (see Table 2) reported their youngest child had or would receive the recommended vaccines.

By completing the first few questions of the survey (Table 2), parents reported their attitudes regarding the importance and safety of vaccines. The overall response was positive in either believing in vaccines' safety or importance to their children. The majority of the parents strongly agreed (91%) in the importance of vaccines in protecting their children from getting serious diseases. As well as about two-thirds of them strongly agreed that immunizations were very important for keeping their children healthy. Similarly, 82% of the parents strongly agreed that the benefits of vaccines outweighed the risks of vaccines. Stressing on herd immunity, when the parents were asked about the importance of vaccines in preventing the spread of diseases in the community, (81%) of them showed strong agreement. Vaccine safety was strongly agreed by most of the parents (82%).

Table 3 showed the attitudes and behaviors regarding the vaccination number of parents surveyed. On the one hand, most parents (75%) were comfortable or somewhat comfortable with the number of vaccines that children receive in their first two years of life. On the other hand, 23% of the parents were neutral in their response or strongly agreed that they were concerned about too many vaccines, potentially damaging a child's immune system. When asked what the maximum number was of vaccines they were comfortable to provide to their child in one visit, the most common response was 1 to 2 (42.2%), followed by 3 to 4 (33.6%), and “whatever the doctor recommends” (22.5%).

This part of the survey showed concerns regarding vaccines, as outlined in Table 4. Parents were told to choose an option out of eight. The options were known as potential vaccines related concerns. Parents were also given the opportunity of stating that they had no vaccine-related concerns. More than half of the parents (55%) reported vaccine was painful and causing fever as the most common concern. About one-fourth of the parents reported that none of the eight options listed were of concern to them. Too many vaccines in one doctor's visit were another concern to be reported by 16% of the parents. Only 13 parents (3%) reported less common concerns including vaccines may cause learning disabilities and vaccines may cause chronic diseases, and others showed in Table 4.

**Figure 2:**
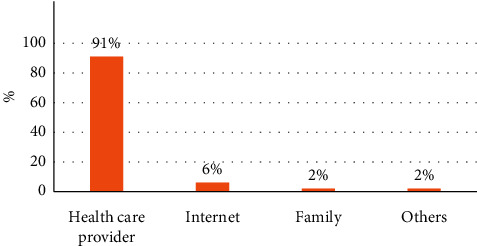
The most important sources of information regarding vaccinations reported by the surveyed parents.

In the last part of the survey, parents were asked about the most important information source. As Figure 2 showed, health care provider was the most common source reported by the parents (91%). Other sources were reported including Internet (6%), family (2%), and other sources constitute about (1%).

## 5. Discussion

Generally, the surveyed parents in Palestine reported positive attitudes toward vaccines. Most of them felt that vaccines were important to their children's health and were confident in vaccine safety. Finding that almost all the surveyed parents reported their children given the recommended vaccines is very encouraging and points to the high awareness level between the Palestinian parents toward the importance of vaccines as well as giving an optimistic view of high vaccines coverage among Palestinian children.

The importance of vaccines in protecting against serious diseases was obvious as shown by more than 90% agreement of our surveyed parents. However, one-tenth (10%) of the parents were concerned regarding vaccines' importance. This result is encouraging when compared to other studies including the Tawam Hospital Survey that showed “20% of the parents surveyed were not fully confident in the importance of vaccines” [9]. Putting things together, the positive trend among the Palestinian parents (more than two-thirds of them) was also clear in the agreement regarding vaccines benefits, the importance of keeping children healthy as well as keeping the herd immunity up to the desired level.

Vaccines safety has been a point of conflict since the early time when vaccines were administered to the public. Additionally, it is not surprising that concerns about vaccine safety have been associated with vaccine refusal by parents [12–15]. Our study showed about one-fifth of the surveyed parents had concerns linked to vaccine safety. However, this percentage was higher among other studies including Kennedy et al. study (about 25% had concerns regarding vaccines safety). However, the percentage was similar to the Twam Hospital Study, which showed about 21% of the surveyed parents had vaccines safety issues and concerns. Recent work by Freed et al. for parents of young children showed that confidence in the importance of vaccines to protect children's health was high, yet concerns about issues such as potential adverse effects were common [16].

High vaccination-coverage levels reinforce the overall confidence in vaccine safety among Palestinian children. Obviously, it showed the extremely low proportion of children nationally who have received no vaccines. As mentioned previously, most parents also believe that vaccines are important, but with one-fourth of them were not fully confident in the safety of vaccines, it is of a crucial issue to go for the education regarding the benefits of vaccines and the potential dangers of the diseases they prevent.

By going through vaccines concerned reported by the surveyed parents, it was found that more than half of the parents had concerns regarding vaccines. The most ones reported were pain and fever following shots. To a lesser extent, one-fourth of the surveyed parents had concerns regarding the number of vaccines in the first two years of life; also, there was concern that the immune system could be weakened by too many vaccines. So the number of vaccines constitutes a bothersome for some parents.

This finding is not new. It is consistent with the results of both past and recent research on parental vaccine attitudes [4, 16]. For example, in a telephone survey of parents of young children conducted in 1999, Gellin et al. reported that parents were supportive of vaccines overall, yet approximately one-fourth of them were concerned about the number of vaccines children receive and the perceived negative effect of vaccines on a child's immune system [4]. These findings emphasize that parents' vaccine-related concerns vary and, as a consequence, effective communication will likely need to be responsive to the series of concerns that individual parents are most likely to feel and consider. Moreover, the above findings highlight the need to address parents' specific questions and concerns about vaccines, even among parents whose overall confidence in vaccines is high.

There are different approaches studied to deal with vaccines concerns reported by parents. It should be focused on well education and reassurance given to parents during the scheduled immunization visits discussing the harmless effect of some potential side effects that may occur postvaccination. Interventions such as breastfeeding, sweet-tasting solutions, pacifiers, distraction, and topical local anesthetics have been associated with decreased pain and crying time for infants [17–20]. Parental stress has been found to be significantly related to an infant's crying time, so by doing the previous measures, infants and also the parents may get benefit [20].

This study as well as others showed that parents reported health care providers as the most important source of information when making decisions about vaccines [4, 21]. This will spot the light on the importance of teaching the health care provider the best way of communication, spending more time with the parents discussing the importance of vaccines and, as mentioned above, the possible harmless side effects that they may encounter postimmunizations.

To summarize, making the immunization encounter less painful for infants and less stressful for parents may also help to reduce the number of concerns parents have about vaccines in general. Teaching parents and empowering them to use evidence-based, soothing interventions that they can offer their children during vaccine administration might help parents have more of a sense of control and engagement in each immunization encounter.

## 6. Conclusion

Our survey revealed that although parental confidence in vaccine safety is high, several vaccine-related concerns, such as pain from vaccine administration, postvaccination fevers, and the number of vaccines given at once, were common among parents of young children. Health care providers continue to be parents' most trusted source of vaccine information and advice. To maintain and improve the success of childhood vaccines in preventing disease, a holistic approach is needed to address these issues in an ongoing manner. Understanding that parents have different questions, concerns, and information needs is the first step. Listening and responding in ways and with resources that address their specific questions and concerns, along with the utilization of comfort measures that can make immunization visits less stressful for both child and parent, might help parents make more informed vaccination decisions.

Here in Palestine, we intend to do scientific lectures in different cities for health care providers regarding the best of communications with parents during the vaccination visits. It should focus on the studied concerns relieving parental stress by evidence-based approach and facts. We may consider repeating the survey after 2 years from ending the plans discussed earlier and see the differences in terms of concerns, percentages of parents who consider the vaccines unsafe and not important for their children.

### 6.1. Strength of the Study

To our knowledge, this study is the first study done to explore the attitudes of northern Palestinian parents about vaccination. And it touches a very important public health issue.

### 6.2. Limitations

This study was conducted on parents in North Palestine who have specific socioeconomical characteristics which although share common cultural backgrounds, traditions, and thoughts, may be not totally representative of all the society for this age group in Palestine. It might be better to conduct the study on a larger and more diverse group that represents all areas in Palestine.

A possible bias in this study is related to sources of information regarding vaccinations which was shown mostly from the primary care provider. Parents would choose this category because the interview was conducted in primary care centers and emergency departments which could be different if the interview was conducted in places not related to medical facilities.

## Figures and Tables

**Table 1:** Demographics date of the surveyed parents.

Factor	Number surveyed	Percentage surveyed (%)
Age		
18–24 years	80	16
25–34 years	270	56
>34 years	130	28
Education		
School or less	153	32
University degree	240	50
Postgraduate degree	87	18
Total	480	100

**Table 2:** Attitudes and behaviors regarding vaccination importance and safety of parents surveyed.

Item	A (N)	B (N)	C (N)
My youngest child had or would receive all the recommended vaccines	8	6	466
My child could get a serious disease if he or she were not vaccinated	437	31	12
It is important to vaccinate my child in order to prevent the spread of disease in my community	389	55	36
The benefits of vaccines outweigh the risks of vaccines	408	48	24
Vaccines are safe for children	394	62	24
Immunizations are very important for keeping children healthy	360	92	28

A: Strongly/Somewhat agree. B: Neither agree nor disagree. C: Strongly/Somewhat disagree. N: Number of parents surveyed.

**Table 3:** The attitudes and behaviors regarding the vaccination number of parents surveyed.

Item	Number (N)	Percent (%)
I am concerned that my child's immune system could be weakened by too many vaccines		

Strongly/somewhat agree	10	2
Neither agree nor disagree	58	13
Strongly/somewhat disagree	412	85

How comfortable are you with the recommended number of childhood vaccines in the first 2 years of a baby's life?		

Comfortable	297	62
Somewhat comfortable	62	13
Not comfortable	121	25

What is the maximum number of vaccines you are comfortable to provide to your child in one visit?		

One or two	221	46
Two or three	148	31
Whatever the doctor recommends	111	23

**Total**	**480**	**100**

**Table 4:** Concerns regarding vaccines reported by the surveyed parents.

Concerns regarding vaccines	Number	Percent (%)
Painful and causing fever	264	55
Too many vaccines in one doctor's visit	77	16
Vaccines may cause learning disabilities (such as autism)	2	3
The ingredients in vaccines are unsafe	1	
Vaccines are given to children to prevent diseases that are not serious	4	
Vaccines are given to children to prevent diseases that they are not likely to get	4	
Vaccines may cause chronic disease (such as diabetes, asthma)	3	
No concerns	125	26
Total	480	100

## Data Availability

The data used to support the findings of this study are available from the corresponding author upon request.

## Appendix

### Vaccinations Attitudes Survey

(1) How old are you?
  - 18—24.
  - 25—34.
  - 35 and above.
(2) Educational level.
  - School or less.
  - University degree.
  - Postgraduate degree.
(3) My youngest child had or would receive all the recommended vaccines.
  - Strongly/somewhat agree.
  - Neither agree nor disagree.
  - Strongly/somewhat disagree.
(4) My child could get a serious disease if he or she were not vaccinated.
  - Strongly/somewhat agree.
  - Neither agree nor disagree.
  - Strongly/somewhat disagree.
(5) It is important to vaccinate my child in order to prevent the spread of disease in my community.
  - Strongly/somewhat agree.
  - Neither agree nor disagree.
  - Strongly/somewhat disagree.
(6) The benefits of vaccines outweigh the risks of vaccines.
  - Strongly/somewhat agree.
  - Neither agree nor disagree.
  - Strongly/somewhat disagree.
(7) Vaccines are safe for children.
  - Strongly/somewhat agree.
  - Neither agree nor disagree.
  - Strongly/somewhat disagree.
(8) Immunizations are very important for keeping children healthy:
  - Strongly/somewhat agree.
  - Neither agree nor disagree.
  - Strongly/somewhat disagree.
(9) I am concerned that my child's immune system could be weakened by too many vaccines.
  - Strongly/somewhat agree.
  - Neither agree nor disagree.
  - Strongly/somewhat disagree.
(10) How comfortable are you with the recommended number of childhood vaccines in the first 2 years of a baby's life?
  - Comfortable.
  - Somewhat comfortable.
  - Not comfortable.
(11) What is the maximum number of vaccines you are comfortable to provide to your child in one visit?
  - 1 or 2.
  - 3 or 4.
  - Whatever the doctor recommends.
(12) What are your concerns regarding vaccines?
  - Painful and causing fever.
  - Too many vaccines in one doctor's visit.
  - Vaccines may cause learning disabilities (such as autism).
  - The ingredients in vaccines are unsafe.
  - Vaccines are given to children to prevent diseases that are not serious.
  - Vaccines are given to children to prevent diseases that they are not likely to get.
  - Vaccines may cause chronic diseases (such as diabetes, asthma).
  - No concerns.
(13) What is the most important sources of information that have helped you make decisions about your youngest child's vaccinations?
  - Health care provider, such as a doctor or nurse.
  - Family
  - Internet.
  - Others.
